# Collaborative Perception—The Missing Piece in Realizing Fully Autonomous Driving

**DOI:** 10.3390/s23187854

**Published:** 2023-09-13

**Authors:** Sumbal Malik, Muhammad Jalal Khan, Manzoor Ahmed Khan, Hesham El-Sayed

**Affiliations:** 1 College of Information Technology, United Arab Emirates University, Abu Dhabi 15551, United Arab Emirates; 201990107@uaeu.ac.ae (S.M.); 201990067@uaeu.ac.ae (M.J.K.);; 2 Emirates Center for Mobility Research (ECMR), United Arab Emirates University, Abu Dhabi 15551, United Arab Emirates

**Keywords:** collaborative autonomous driving, collaborative perception, fusion, evolved RSU, C-V2X

## Abstract

Environment perception plays a crucial role in enabling collaborative driving automation, which is considered to be the ground-breaking solution to tackling the safety, mobility, and sustainability challenges of contemporary transportation systems. Despite the fact that computer vision for object perception is undergoing an extraordinary evolution, single-vehicle systems’ constrained receptive fields and inherent physical occlusion make it difficult for state-of-the-art perception techniques to cope with complex real-world traffic settings. Collaborative perception (CP) based on various geographically separated perception nodes was developed to break the perception bottleneck for driving automation. CP leverages vehicle-to-vehicle and vehicle-to-infrastructure communication to enable vehicles and infrastructure to combine and share information to comprehend the surrounding environment beyond the line of sight and field of view to enhance perception accuracy, lower latency, and remove perception blind spots. In this article, we highlight the need for an evolved version of the collaborative perception that should address the challenges hindering the realization of level 5 AD use cases by comprehensively studying the transition from classical perception to collaborative perception. In particular, we discuss and review perception creation at two different levels: vehicle and infrastructure. Furthermore, we also study the communication technologies and three different collaborative perception message-sharing models, their comparison analyzing the trade-off between the accuracy of the transmitted data and the communication bandwidth used for data transmission, and the challenges therein. Finally, we discuss a range of crucial challenges and future directions of collaborative perception that need to be addressed before a higher level of autonomy hits the roads.

## 1. Introduction

The evolution of computing, communication infrastructure, sensor technologies, and artificial intelligence has pushed the limits for a variety of industries, such as healthcare, autonomous driving, and many others. Autonomous driving (AD), with its capability for safe and dependable operation, can significantly decrease road accidents, relieve traffic congestion, and reduce fuel consumption by transferring control of the driving task from human drivers to autonomous vehicles (AVs).

The Society of Automotive Engineers (SAE) [1] categorizes AVs into six levels of autonomy. The majority of currently available commercial features fall under level 2 and level 3, referred to as advanced driving assistance systems (ADAS). These systems have been successful in environments that are well-structured and controlled such as highways. However, the complexities of navigating in urban areas filled with mixed traffic, unpredictable events, and vulnerable road users, such as pedestrians and cyclists, make it difficult for classical AVs to operate safely, efficiently, and without congestion. Thus, the push is towards a fully level 5 AD to address these challenges. Both the industry and research communities are actively working on solutions, including collaborative autonomous driving, to attain a higher level of autonomy. Collaborative autonomous driving (CAD) makes it possible for connected and autonomous vehicles (CAVs) to interact with one another, with the infrastructure of the road, or with other road users, such as pedestrians and cyclists carrying mobile devices, aiming to enhance traffic efficiency and road safety [2]. As a result, CAD has gained in popularity over the last few years and is recognized as a potential solution to many transportation issues.

The ongoing advancements in sensor technology for AVs have become a crucial factor in increasing the range and accuracy of their sensing abilities, as well as ensuring the necessary information required while driving, such as road conditions, traffic signs, and obstacle information, is readily available. However, maintaining real-time situational awareness of the environment for AVs above level 3 is difficult to achieve solely through the vehicle’s perceptual capabilities, due to the limited environmental perception information, computing, and communication resources.

Therefore, to overcome the limitations of perception and computation in autonomous driving above level 3, a collaborative perception (CP) system needs to be developed by incorporating advanced sensing technology, edge computing, 5G communication, and other technologies. This will increase perception accuracy, expand the perception range, and decrease delay. Moreover, it will provide AVs with more accurate and comprehensive environmental information in real-time, laying the groundwork for the implementation of higher-level autonomous driving. Additionally, CP can reduce the need for onboard sensors, lower the cost of autonomous vehicles, and accelerate the commercialization of autonomous driving at higher levels.

In this article, we comprehensively study and review the transition from classical perception to collaborative perception (Section 2), perception creation at different levels (Section 2.1 and Section 2.2), analysis of communication technologies (Section 3.1), collaborative perception message-sharing models and a range of crucial challenges (Section 3.2), and future directions of collaborative perception (Section 4) that need to be addressed before a higher level of autonomy hits the roads.

## 2. Transition from Classical Perception to Collaborative Perception

AVs comprise three essential subsystems: perception, planning, and control. Perception is one of the crucial systems of AVs that refers to the ability of an autonomous vehicle to acquire data from various sources and create an understanding of its surroundings such as the distance between the vehicle and an obstacle, the kinds of objects that are close to the vehicle, detection of traffic signs, comprehension of traffic conditions, lane recognition, comprehension of curves, etc. AVs process information from a variety of sources to construct perception, and these sources can broadly be divided into on-board sources (LiDAR, radar, cameras, etc.) and external information sources (neighboring vehicles, evolved roadside units (eRSUs), infrastructure) [3].

The transition from perception to collaborative perception in AD is a shift from a traditional approach to perception, where a single vehicle senses and interprets the environment, to an approach that involves multiple vehicles working together to sense and interpret the environment. AVs equipped with heterogeneous sensors sense their immediate surroundings and execute control operations and intelligent transport system (ITS) services. These local perceptual cues alert the AVs to the presence of various objects in the driving environment, including other autonomous vehicles, classical vehicles, trees, cyclists, pebbles along the road, etc. However, each sensor’s perceptual abilities are constrained to a specific detection range and field of view. Moreover, these capabilities can also be hampered by the existence of objects (obstructions) in the field of view, sensors’ blind spots, adverse weather conditions, environments that are obscured, and sensitivity to ambient light and temperature, among other factors. These restrictions severely reduce the perceptive capabilities of AVs and these may cause catastrophic consequences with detrimental effects on both their safety and driving performance. The comparison of the characteristics of different sensors mounted on the autonomous vehicle is presented in Table 1. Readers are highly encouraged to look into [4,5] for the detailed specifications of each sensor.

It is possible for autonomous driving systems to be affected by adverse environmental conditions such as rain and snow. For instance, water droplets on lenses, obstructions in the fields of vision, and scatterings of signals may reduce the performance of on-board and on-road deployed sensors such as cameras, LiDARs, and radars. In addition, it is possible for precipitation to affect the signals and cause false positives or false negatives by creating reflections and other interferences. Therefore, multi-modality solutions [6] have been used to address these issues posed by environmental effects.

By integrating large-scale training data and deep learning approaches with improvements in sensor and computing technologies, classical perception systems have recently undergone a remarkable degree of evolution. Despite these noteworthy developments, the limited line of sight of onboard sensors remains an immovable obstacle, and the performance of the perception system is easily impacted by external factors such as weather and road conditions. Furthermore, the fact that each vehicle senses the surrounding world based on its own local perception sensors, that is, individual perception, is a major factor limiting the performance of autonomous driving in terms of perception. Individual perceptual capacity is, however, restricted, and as a result, the restricted field of view, missing modalities, sparse sensor data, and other adverse circumstances reduce perception. However, that pledge of increased safety is compromised by the fact that a vehicle’s sensors have a limit on the degree and range of its perception. Therefore, for the dynamics of complex settings, these cutting-edge techniques for vehicle perception might not be adequate. Additionally, the perception for specialized use cases, such as platooning, remote driving, etc., is distinct and necessitates additional infrastructure and sources of information.

Ideally, in all complex settings, the understanding of the environment should be precise and in real-time. Therefore, to cope with the challenges of classical perception, there is a dire need to introduce an evolved version of collaborative extended perception that should address the challenges hindering the realization of level 5 autonomous driving by extending the vision and enriching the understanding of AVs.

CP naturally draws on two fundamental components: communication and perception [7]. By exchanging information with neighboring vehicles and infrastructure, CP enables AVs to circumvent perceptual constraints such as occlusion and a limited field of view. In this approach, vehicles collaboratively communicate sensing and perceptual data in an effort to widen their field of view, enhance precision, and eliminate blind spots. Therefore, augmented situational awareness without incurring any additional cost can be summed up as collaborative perception’s key benefit. For instance, cooperative perception enables a vehicle to have a broad field of view and line of sight since the sensing region can be extended to encompass the union of the sensing regions of all CAVs, infrastructure, and smart towers. Resultantly, the cooperative vision would allow a vehicle to make the optimal decisions proactively far earlier than it otherwise could have been able to do. This has a clear advantage for users since cooperative perception sensors and radio equipment are cheaper than traditional long-range sensors.

Collaborative perception can be created at different levels such as vehicles and towers. In what follows next, we discuss the two main approaches to collaborative perception creation in detail.

### 2.1. Perception Creation at the Vehicle Level

Industry and academia are paying attention to a new technology referred to as vehicle-to-vehicle (V2V) collaborative perception, which involves the real-time sharing of sensor data between vehicles leveraging V2V communication. In this connection, the cooperative/collective perception service standardization and cooperative perception messages (CPMs) have also been introduced by the ETSI (European Telecommunications Standards Institute) [8]. Collaborative perception at the vehicle level considers the input from a variety of viewpoints to reconcile a global view of the environment that draws on the different viewpoints of each vehicle. The field of view of the individual vehicle is extended by the shared and fused information since its sensing zone is effectively expanded by aggregating the perception data from all nearby vehicles. The potential to increase driving safety, particularly in situations where accidents are likely to happen, is provided by the shared perspective, which also communicates the intentions and path planning of neighboring vehicles. Additionally, CP has the potential to significantly enhance the accuracy of the environmental perception, thereby enabling better driving decisions such as overtaking, avoiding unexpected or hidden obstacles, or early lane changes against lane drop, ultimately improving traffic safety and efficiency.

**Figure 1:**
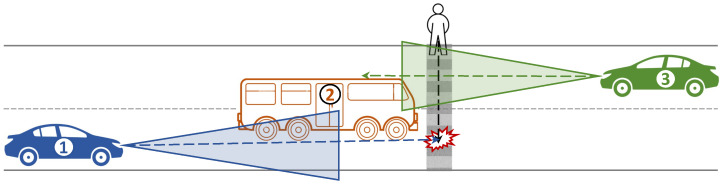
Illustration of a hazardous situation caused by occlusion.

Consider the traffic scenario illustrated in Figure 1, where a pedestrian is crossing the road while the traffic light is green for them. However, once the pedestrian reaches the middle of the road, the light changes to green for Vehicle 1 and Vehicle 2 (bus). The bus, however, obscurs the view of Vehicle 1 and makes it difficult for it to detect the pedestrian. This increases the risk of Vehicle 1 hitting the pedestrian even if it applies the brakes. To address this safety concern, a potential solution, CP leveraging V2V communication can be used, where both Vehicle 1 and Vehicle 3 (moving in the opposite direction) share their perceptions. Figure 1 shows that Vehicle 3 can detect the pedestrian, so sharing Vehicle 1’s perception with Vehicle 3 would extend the sensing range and enhance safety, so Vehicle 1 can avoid hitting the pedestrian.

There are several approaches to creating collaborative perception such as (i) image-based collaborative perception [5] and (ii) point cloud-based collaborative perception [9]. The perception created collaboratively by the neighboring multiple vehicles can then be applied to a variety of downstream modules, including localization, mapping, path planning, tracking, semantic segmentation of 3D scenes, detection of blind areas, and recognition of 3D objects.

In what follows next, we review the latest research to create perception at the vehicle level:

The advancement of telecommunication technologies has opened up new possibilities for improving autonomous driving in dangerous or emergency scenarios through cooperative perception using V2V communications. Cui et al. [10] introduced a groundbreaking approach called COOPERNAUT, which employed an end-to-end learning model for vision-based cooperative driving. This innovative model transformed LiDAR data into concise point-based representations that could be exchanged as messages between vehicles using realistic wireless channels. In order to assess the effectiveness of the model, the researchers developed AUTOCASTSIM [11], a driving simulation framework augmented with network capabilities, and conducted experiments involving accident-prone scenarios. The results obtained from AUTOCASTSIM demonstrated that the cooperative perception driving models outperformed egocentric driving models, achieving a remarkable 40% improvement in the average success rate in challenging driving situations. Furthermore, these models required only five times less bandwidth compared to the previous V2VNet [12] approaches. The utilization of V2V communication in CP facilitates the exchange of deep learning-based features among vehicles, and reinforcement learning-based vehicular edge computing (VEC) [13]. However, existing cooperative perception algorithms typically assume flawless communication and overlook the consequences of lossy communication (LC), which is prevalent in real-world scenarios in feature sharing. In order to address this issue, Li et al. [14] conducted a study on the influence of LC on CP and proposed a novel solution to mitigate these effects. Their proposed approach encompassed an LC-aware repair network (LCRN) and a V2V attention module (V2VAM) incorporating intra-vehicle attention and uncertainty-aware inter-vehicle attention. By employing a point cloud-based 3D object detection on the publicly available OPV2V [15] dataset, they effectively demonstrated the efficacy of their approach. Furthermore, the results revealed that the proposed method significantly enhanced detection performance when operating under lossy V2V communication. Notably, the approach achieved a noteworthy improvement in average precision compared to the most advanced cooperative perception algorithms, substantiating its ability to effectively mitigate the adverse impact of LC and enhance the interaction between the EGO vehicle and other vehicles.

The utilization of multi-agent collaborative perception has the potential to greatly enhance perception performance by facilitating the exchange of complementary information among agents through communication. However, this approach inevitably introduces a fundamental trade-off between perception performance and communication bandwidth. To address this bottleneck, Zhong et al. [16] proposed Where2comm, a novel framework for the collaborative perception that emphasizes efficient communication. Where2comm leveraged a spatial confidence map at each agent to enable practical compression, guiding agents in determining what information to communicate, to whom, and whose information to aggregate. By providing spatially sparse yet crucial features to support other agents while also requesting complementary information through multiple rounds of communication, Where2comm achieved a significantly improved trade-off between perception performance and communication bandwidth. However, a limitation of their work is its focus on spatially critical areas, and it would be beneficial in future research to expand this concept to the temporal dimension by identifying critical time stamps. Additionally, exploring the timing of communication could further reduce costs. Furthermore, the application of pragmatic compression and emergent communication methods holds the potential for enhancing collaborative perception. Current multi-agent perception systems assume that all agents use the same model with identical parameters and architecture. However, when different perception models are employed, the performance can suffer due to inconsistencies in their confidence scores. To address this issue, Xu et al. [17] presented a model-agnostic multi-agent perception framework that mitigates the negative impact of model discrepancies without sharing the actual model information. Their approach involved a confidence calibrator capable of rectifying biases in prediction confidence scores. Each agent independently performed this calibration process using a standardized public database, ensuring the protection of intellectual property. The experiments conducted underscored the importance of model calibration across diverse agents, with the results demonstrating that the proposed framework significantly improved the baseline performance of 3D object detection in the context of heterogeneous agents.

With the increasing prevalence of machine learning techniques in autonomous vehicles, the perception subsystem has gained significant attention. Consequently, Yang et al. [18] proposed the concept of developing a machine learning-enabled cooperative perception system for CAVs. In order to achieve cooperative perception, the authors emphasized the advantages of sharing feature map data between vehicles rather than raw sensor data. This approach offers superior privacy protection and allows for flexibility in the amount of data to be transmitted. To facilitate cooperative perception, the authors utilized high-speed millimeter-wave (mmWave) communications for the fast and reliable transmission of feature maps. However, the design of a feature map-based cooperative perception system for CAVs presents several technical challenges that must be addressed to ensure reliability and practicality. The authors identified research challenges including feature map compression, feature map selection, mmWave communications, and vehicular edge computing. While these challenges pave the way for more advanced solutions in cooperative perception for CAVs, the authors acknowledge that further work is required to overcome them. In order to address the issue of transmitting large amounts of data, current CP solutions utilize feature maps generated by convolutional neural network (CNN) models, as intermediate data. However, these feature maps are often too large to be effectively transmitted using existing V2X technology. To tackle this challenge, Guo et al. [19] introduced an innovative approach called Slim-FCP, which significantly reduced the size of the transmitted data. This approach employed a channel-wise feature encoder to eliminate irrelevant features, resulting in an improved compression ratio. Additionally, Slim-FCP incorporated an intelligent channel selection strategy that selectively transmitted representative channels from the feature maps. To assess the efficacy of Slim-FCP, the authors introduced a metric called the recall-to-bandwidth (RB) ratio, which quantitatively measured the impact of available network bandwidth on object detection recall. Experimental results demonstrated that Slim-FCP achieved a remarkable 75% reduction in transmission data size compared to the best state-of-the-art solution, with only a minor decrease in object detection recall.

### 2.2. Perception Creation at the Tower Level

AVs require a deep understanding of their surroundings and an accurate perception of the environment to execute safe and efficient operations on the road. In this context, the concept of smart towers or evolved roadside units (eRSUs) is considered a core component in realizing AD in the intelligent environment. The eRSUs located on the road are equipped with heterogeneous sensors, computing, and communication infrastructure. These sensors gather data about the surroundings and create a rich and detailed perception of the environment in real-time. This extended perception is then shared and communicated with the CAVs, enabling them to receive real-time information about traffic conditions, weather, and other factors that may affect their operation. This information can be used to optimize the vehicle’s path planning, speed, and behavior to ensure safe and efficient operation. However, it is challenging to predict the margin of uncertainty within seconds when AVs are analyzing various traffic patterns, road conditions, and other factors such as object detection, localization, trajectory planning, and decision-making. Additionally, it is possible to achieve faster data transmission and reduced latency using next-generation mobile network technologies such as 5G (5th generation) [20] and 6G (6th generation) [21]. Therefore, it can reduce the time necessary to measure errors and uncertainties in a variety of traffic conditions, potentially reaching millisecond (ms) levels. Given that, the communication performance impacts the collaborative perception process during platooning, particularly when vehicles are interacting at intersections or at different complex road segments under communication disturbance [22,23].

**Figure 2:**
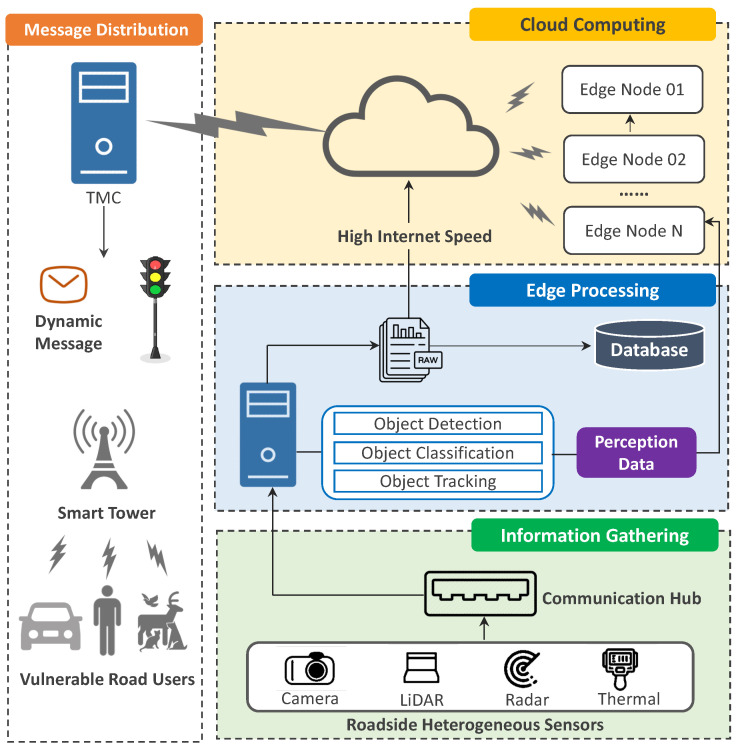
Architecture of the infrastructure-based perception system [24].

Zhengwei et al. [24] presented the architecture of an infrastructure-based perception system (see Figure 2). The system comprises four main components: (i) Information Collection—roadside infrastructures are equipped with heterogeneous sensors that detect the surroundings and send the gathered data to a communication hub that then transmits it to the roadside server for additional analysis; (ii) Edge Processing—due to the constrained bandwidth available for transmitting extensive amounts of raw data, such as point cloud datasets, the information obtained from roadside sensors can be processed on a local server located at the edge (roadside). Typically, there are three primary stages involved in processing the initial sensing data during this phase: pre-processing, object perception, and storage; (iii) Cloud Computing—typically, roadside equipment generates perception data as a result of the large volume of raw data. These perception data are then wirelessly transmitted to the Cloud using technologies such as cellular networks or wireless local area networks. In systems with high-speed internet, raw data can also be directly sent to the Cloud for processing. In the case of multi-node perception systems, where the environment is simultaneously perceived from different locations, spatiotemporal information assimilation and synchronization require the careful consideration of time alignment (including delay compensation) and object association; (iv) Message Distribution—perception information, along with advisory or actuation signals, can be disseminated to road users through two main methods based on their connectivity status. For conventional road users without wireless connectivity, this information can be delivered to roadside end devices such as dynamic message signs (DMS) or traffic signal displays via the traffic management center (TMC). For connected road users with wireless communication capabilities, customized information, such as surrounding objects and upcoming signal timings (SPaT), can be accessed to facilitate various cooperative driving automation applications including cooperative eco-driving.

**Figure 3:**
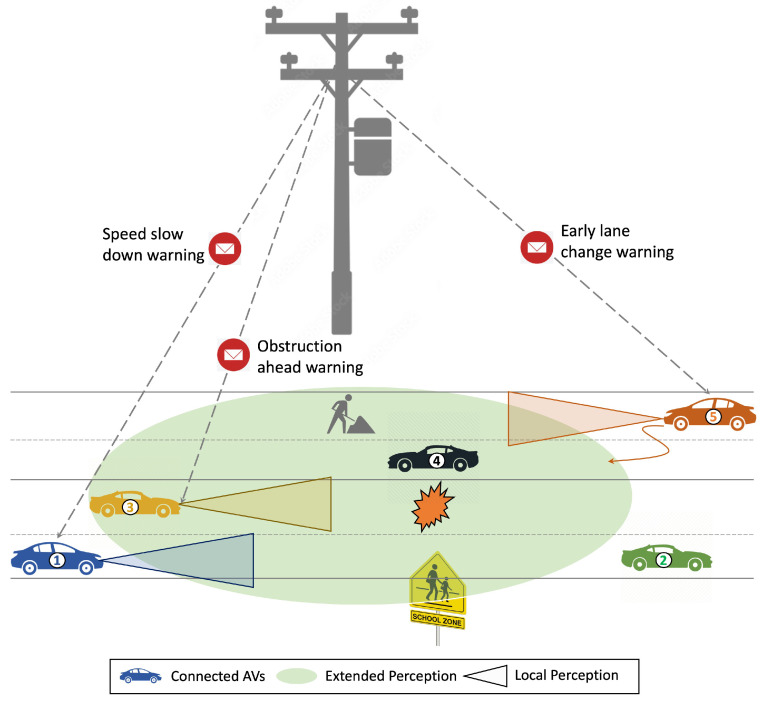
Infrastructure for the vehicle collaborative perception.

Consider Figure 3 as an example of a smart tower located on a road equipped with heterogeneous sensors, computing and communication infrastructure, and an artificial intelligence toolbox that creates the extended perception of the road segment represented in a green oval. In the case of Vehicle 1, it has limited perception and cannot detect that the school zone is a few meters ahead. However, the tower with its extended perception detected it earlier and sends a warning to Vehicle I to slow down. Similarly, in the case of Vehicle 2, the smart tower detects that a lane is closed due to construction, it sends an alert to the AV to notify it of the closure, resultantly enabling the vehicle to make an early lane change. Furthermore, in the case of Vehicle 3, the tower detects the obstruction on the road and alerts the vehicle in advance to take proper action. Overall, the smart tower perception system provides the AVs with a more complete and accurate picture of the surrounding environment, allowing them to make more informed decisions and operate more safely and efficiently.

In what follows next, we review the latest approaches to create perception at the tower level:

To realize collaborative perception, the eRSUs make use of their high-tech sensors for collecting and sharing data in the form of images, videos, point clouds, etc., with the vehicles in the road segment or region. Data sharing can be achieved through V2I or V2I2V communication technology. Depending on the collaboration levels, i.e., early, intermediate, and late, the data can be in the form of raw sensory data or processed features in the form of object data [25,26]. Chen et al. [27] proposed a solution approach to enhance the accuracy of autonomous vehicle decision-making through multi-vehicle cooperative perception using LiDAR 3D point clouds. The proposed system outperformed the traditional perception creation approach and could be transmitted via existing vehicular networks. In another study, Arnold et al. [28] presented a cooperative 3D object detection in autonomous vehicles. They compared early fusion and late fusion and showed that early fusion outperformed late fusion while requiring higher communication bandwidth. Finally, they concluded that cooperative perception can significantly improve object detection recall compared to single-point sensing. Driving automation, a significant advancement in modern transportation systems in terms of safety, mobility, and sustainability, heavily relies on 3D object detection. However, most state-of-the-art object detection methods using point clouds are designed around a single onboard LiDAR, which is inherently limited by its range and susceptibility to occlusion, particularly in dense traffic scenarios. To address this, Bai et al. [29] developed PillarGrid, a novel cooperative perception framework that combines information from multiple 3D LiDARs (both onboard and roadside) to enhance situational awareness for connected and automated vehicles (CAVs). PillarGrid comprises four key components: (i) cooperative preprocessing of point clouds, (ii) pillar-wise voxelization and feature extraction, (iii) grid-wise deep fusion of features from multiple sensors, and (iv) augmented 3D object detection using a convolutional neural network (CNN). To facilitate model training and testing, a unique cooperative perception platform was developed. Extensive experimentation demonstrated that PillarGrid surpasses other single-LiDAR-based 3D object detection methods by a significant margin, excelling in both accuracy and range. To facilitate collaborative intelligence between vehicles and roads, V2I communication has emerged as a potential solution. Duan et al. [30] introduced RGB-PVRCNN, an environment perception framework aimed at enhancing the situational awareness of AVs at intersections through the utilization of V2I communication technology. The framework integrated the vision-based features derived from PVRCNN while employing the normal distributions transform point cloud registration algorithm both onboard the vehicles and on the roadside. This enabled the determination of AV positions and the creation of a local map. Detected objects from the roadside multi-sensor system are then transmitted back to the AVs, enhancing their perception capabilities. This augmentation benefits path planning and improves traffic efficiency at intersections. Field-testing results demonstrated the effectiveness of the proposed method in expanding the environmental perception range and ability of AVs at intersections. Moreover, it outperformed both the PointPillar algorithm and the VoxelRCNN algorithm in terms of detection accuracy.

In another study by Aoki et al. [25], a cooperative perception scheme was developed leveraging deep reinforcement learning to enhance object detection accuracy in AVs. The scheme significantly improved accuracy by up to 12% compared to the baseline protocol, reduced network load, and improved communication reliability. In [26], the authors proposed a roadside perception unit that combined sensors and roadside units for infrastructure-based cooperative perception to enhance V2X communication for autonomous driving. The AutoC2X software is designed to support the system and the networked roadside perception units allow for a broader perception range. The system and its priority algorithm experimented and concluded that it can deliver messages to vehicles quickly and accurately even under heavy traffic conditions.

Due to a limited global perspective and the constraints of long-range perception capabilities, AD faces significant safety challenges. Achieving level 5 autonomy necessitates vehicle–infrastructure cooperation, which is widely acknowledged. However, there is currently a dearth of real-scenario datasets available for computer vision researchers to address issues related to vehicle–infrastructure cooperation. To expedite computer vision research and innovation in vehicle–infrastructure cooperative autonomous driving (VICAD), Yu et al. [31] introduced the DAIR-V2X dataset. This dataset is a pioneering large-scale, multi-modality, multi-view collection of real-scenario data for VICAD, consisting of 71,254 LiDAR frames and 71,254 camera frames, all of which have 3D annotations. They also introduced the vehicle–infrastructure cooperative 3D object detection problem (VIC3D) that involves collaboratively locating and identifying 3D objects using sensory inputs from both the vehicle and infrastructure. Additionally, they proposed a benchmark framework called time compensation late fusion (TCLF) for the VIC3D task, based on the DAIR-V2X dataset. Along similar lines, Mao et al. [32] released the DOLPHINS, a groundbreaking dataset for collaborative perception in autonomous driving. It offers a large-scale diverse benchmark platform for interconnected autonomous driving. DOLPHINS outperformed existing datasets in six dimensions: incorporating images and point clouds from vehicles and RSUs, covering various scenarios and weather conditions, selecting comprehensive viewpoints, providing a significant scale with annotations and calibrations, featuring high-resolution data, and ensuring extensibility through APIs and open-source codes. Experimental results showed that the raw-level fusion scheme using V2X communication enhances precision and reduces reliance on expensive LiDAR equipment, potentially accelerating the adoption of interconnected self-driving vehicles.

The maturity and capability of algorithms developed for autonomous driving, such as Kalman Filters [33], sensor fusion algorithms [34,35], etc., have improved considerably; however, it is important to note that depending on the specific implementation and technology, these algorithms may differ significantly. Therefore, the collaborative perception algorithms can minimize confusion between the state of the road and the measurement objective, but in order to enhance the algorithms’ robustness and reliability to prevent the confusion, research and development efforts need to be continued, extensive testing must be completed, and algorithms need to be refined. Significant research has been carried out in this area; however, there are several challenges to fully realizing the potential of eRSUs and AVs. From integrating information from multiple sensors to collaborative perception and developing algorithms that can effectively process the data, these challenges are significant and require combined efforts from industry, academia, and city authorities. In what follows next, we highlight some of the crucial challenges that need to be addressed:

- *Integration of multiple sensors:* the need for integrating information from cameras, LiDAR, and radar sensors to create a comprehensive view of the environment;
- *Collaborative perception:* the need for collaboration among autonomous vehicles and eRSUs for creating an extended perception of the environment;
- *Design and development of sophisticated algorithms:* the need for developing algorithms that can effectively process the large amounts of data generated by the sensors, create a collaborative perception of the environment, and communicate the extended perception with AVs;
- *Ensuring safe and efficient operation:* the importance of having accurate and collaborative perceptions of the environment to ensure the safe and efficient operations of autonomous vehicles; and
- *Addressing potential challenges:* the potential challenges that may arise in the development of collaborative perception through the eRSUs and the importance of addressing these challenges to make the extended perception available to all relevant AVs.

Readers are highly recommended to look into the authors’ survey [3,36] for a complete list of the relevant challenges.

## 3. Collaborative Perception Sharing

This section is divided into two subsections. Section 3.1 discusses the V2X communication technologies and Section 3.2 discusses the perception-sharing models.

### 3.1. V2X Communication Technologies

Currently, there are two primary V2X communication technologies in existence, the dedicated short-range communication (DSRC) and cellular vehicle-to-everything (C-V2X). DSRC specifically caters to V2V and V2I communication and offers several advantages, such as high transmission rate, low latency, and support for point-to-point and Moreover, C-V2X communication, exemplified by 5G, provides wide coverage, high reliability, and ample data capacity. It is particularly suitable for vehicle-road cooperation and communication between edge servers. These two communication modes collaboratively meet the diverse application requirements of the IoV environment.

In what follows next, we discuss both communication technologies briefly and provide their comparison in Table 2.

#### 3.1.1. Dedicated Short-Range Communication

ITS-G5 or DSRC, which utilize the IEEE 802.11p protocol, was specifically designed for facilitating communication among vehicles. IEEE 802.11p is an advancement of the IEEE 802.11a protocol, with a particular emphasis on the time domain parameter. This parameter was doubled due to the dynamic nature of the radio channel. ITS-G5 and DSRC share the same frequency spectrum, namely the 5.9 GHz unlicensed band dedicated to vehicular communication. According to [37], IEEE 802.11p employs a 10 MHz frequency bandwidth, which is half the bandwidth of IEEE 802.11a. This narrower bandwidth enhances the signal’s resilience in the face of rapidly fluctuating channels. By incorporating fading effects, it increases the efficiency in a multi-path propagation environment such as a vehicular network. The protocol combines orthogonal frequency division multiplexing (OFDM) with convolution coding to enhance communication. Data rates can range from 3 MHz to 27 MHz, depending on the modulation scheme used (e.g., BPSK, QPSK, and QAM). In terms of channel access, IEEE 802.11p utilizes carrier sense multiple access (CSMA) with collision avoidance, also referred to as enhanced DCF of IEEE 802.11a [38]. This method prevents collisions and improves re-transmissions when dealing with high-density channel acquisition requests. Another notable feature is the capture effect that prioritizes signals with higher strength over previously received signals. This prioritization enhances the protocol’s performance and encourages the active participation of nodes in receiving and decoding signals. DSRC, as outlined in SAE J2735, defines 15 fundamental message types that are necessary for the implementation of the DSRC protocol, ensuring reliable communication for seamless autonomy. Subsequent revisions of this standard include additional message types related to cruise control and other advanced features [39]. Nevertheless, recent research has indicated that DSRC alone is insufficient for effectively supporting dependable and efficient V2X applications, particularly in scenarios involving dense vehicle populations and high-speed vehicle mobility. Under such conditions, communication performance experiences a noteworthy decline [40].

#### 3.1.2. C-V2X Communication

**Figure 4:**
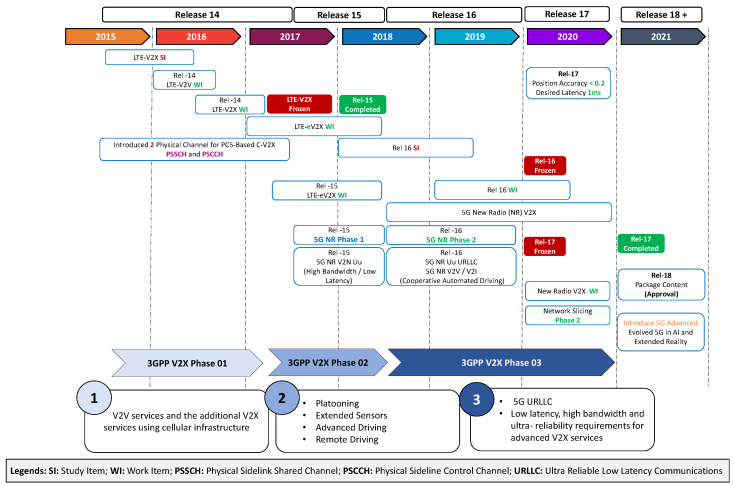
Standardization and evolution of C-V2X [42].

The 3rd Generation Partnership Project (3GPP) [41] standardized C-V2X, encompassing LTE-V2X and 5G-V2X (NR) technologies. This standardization process involves progressive enhancements as can be seen in Figure 4. C-V2X also defines a PC5 interface facilitating direct V2X communication via the sidelink, as well as a Uu interface enabling communication between the terminal and base station through the uplink/downlink [42]. With its extensive network capacity and wide coverage, C-V2X is well-suited for supporting collaborative perception in the internet of vehicles (IoV) environment, enhancing data transmission reliability, reducing transmission delays, and minimizing frequent horizontal switching within the network. Compared to DSRC, C-V2X offers performance advantages such as an increased link budget, improved resilience to interference, and better non-line-of-sight (NLoS) capabilities [43,44]. Choi et al. [45] analyzed this concept based on DSRC and 4G cellular networks, which were deemed insufficient for the large-scale sharing of raw sensor data. They proposed three vehicle network types, including 5G cellular, a modified version of IEEE 802.11ad, and a dedicated new standard that effectively addresses the problem of channel congestion caused by high data transmission volume. These communication methods also enhance the real-time performance of the vehicle networking perception system. Currently, C-V2X is in the phase of actual deployment and application testing, with ongoing system improvements. New radio (NR) V2X, specified in Release 16, serves as a complementary access technology in C-V2X, catering to demanding applications and use cases such as platooning and advanced driving. Furthermore, efforts are actively underway to standardize and test Release-17 [46].

To address the challenges of increased network latency and decreased reliability during high-speed vehicle driving scenarios, Zhu et al. [47] introduced a 5G C-V2X intelligent fusion network technology that incorporates mobile edge computing (MEC). They extensively discussed and outlined the network architecture, deployment strategies, and potential application scenarios. Wei et al. [48] utilized a multi-agent deep reinforcement learning (DRL) algorithm for radio resource selection in C-V2X Mode 4, considering the specific features of cooperative perception. The authors initially developed a model representing a typical scenario involving cooperative perception and communication between vehicles using the C-V2X Mode 4 protocol. Subsequently, they introduced an algorithm to optimize the objective and validated its performance in a simulation environment. The simulation results demonstrated that their algorithm outperformed the original semi-persistent scheduling and random algorithms, leading to enhanced transmission reliability, reduced packet delay, and improved efficiency in cooperative perception scenarios. Fukatsu et al. [49] emphasized the significance of incorporating millimeter-wave communication into cooperative perception, considering the distinct network data transmission needs of autonomous vehicles across varying driving speeds. They observed that the sensor-generated data rate experiences exponential growth as the vehicle speed increases. Moreover, they highlighted the potential of V2V millimeter-wave communication on the 60 GHz band to facilitate cooperative perception in overtaking scenarios even when the speed exceeds 51 km/h. In another study, Fukatsu et al. [50] investigated the sensor rate necessary to achieve specific application functions for urban road scenes in cooperative perception. Their findings indicated that millimeter-wave communication exhibited superior performance in enabling cooperative perception at edge nodes. They validated that at vehicle speeds of 56 km/h and 47 km/h, the required data rates were 12 GHz and 6 GHz, respectively.

### 3.2. Collaborative Perception Message-Sharing Models

A typical pipeline of classical local perception creation involves a network that takes in data gathered by sensors such as LiDAR or RGB cameras, extracts features through an encoder, and produces outputs related to the task at hand. Therefore, to broaden the perspective of the EGO vehicle, collaborative systems exchange pose information and perception data with it and integrate a collaboration module into the perception network. The position information serves to align the transformation and the collaboration module performs specific fusion operations. Based on the message delivery and the stage of collaboration, the CP approach is broadly classified into three types [51]. In what follows next, we briefly discuss these CP message-sharing models.

#### 3.2.1. *Early Collaboration*

It is also referred to as data-level fusion. The collaboration is carried out in the input space, which allows vehicles within the communication range to exchange raw sensory data. It integrates unprocessed measurements from every vehicle to create an all-encompassing viewpoint. The occlusion and long-range problems that arise in single-agent perception could therefore be resolved by each vehicle conducting subsequent processing and finishing perception from a holistic perspective. However, sharing raw sensory data necessitates extensive communication and can quickly overwhelm the communication network with large data loads, which prevents it from being used practically in most circumstances. However, given the availability of high-performance computing and 5G with ultra-reliable low-latency communications (URLLC), early collaboration might be the best option. Additionally, since the raw data shared by the sensors is not processed, it is more reliable, can be utilized for a variety of modules, and is more likely to be free of any data manipulation.

#### 3.2.2. *Intermediate Collaboration*

It is also known as feature-level fusion. It carries out the collaboration in the intermediate feature space by permitting the transmission of the intermediate features generated by each vehicle’s prediction model. Following the fusion of these data, each agent decodes the combined features to produce the perceptual outcomes. In comparison to early cooperation, it offers the best trade-off between detection precision and economical transmission bandwidth. It is currently the most researched and applied fusion method. Recently, several intermediate fusion techniques for V2V CP have been presented. The following techniques are recommended for the readers: OPV2V [15], V2VNet [12], F-Cooper [52], and DiscoNet [53].

#### 3.2.3. *Late Collaboration*

It is also known as decision-level fusion and carries out collaboration in the output space. It encourages the fusion of the perceptual results produced by each vehicle to obtain refinement. Although late collaboration is bandwidth-efficient, it is very susceptible to agent positioning errors and suffers significant estimation errors and noise as a result of inadequate local observation.

In conclusion, there is a trade-off between comprehension, the accuracy of the data, and the communication bandwidth. The choice of collaboration style depends on several important factors such as the driving context and environment, use cases, the availability of high-performance computing, 5G and 6G communications, privacy, the trustworthiness of the shared data, etc. The summary and decision challenges common to all of these models are presented in Table 3.

## 4. Challenges and Future Directions

This section presents the challenges and future directions for collaborative perception in realizing fully autonomous driving.

### 4.1. Data Governance

The collaborative perception either at the vehicle level or infrastructure level (eRSU) uses several sensors and requires processing of the captured data either individually or in a fusion manner. Hence, an environment with heterogeneous data is created for realizing the higher levels of autonomous driving, which asks for common data standards and protocols for communicating the perception between vehicles and eRSUs. In such a data-rich environment where the data is captured by many onboard sensors in the vehicle and on-road sensors in the eRSU, it is becoming difficult to know the ownership of the data and who has the right to access it from where and at what time frame. In addition, the level of trust between vehicles and eRSUs can heavily influence the safety of autonomous vehicles. It is a significant challenge to have trustworthy communication bit-pipes and accurate CP for safe and efficient autonomous driving. However, the decentralized networks i.e., peer-to-peer networks and blockchain-based networks can help in achieving trustworthiness and reliability in the CP of AVs [54]. Furthermore, machine learning models, such as federated learning, can be used to preserve data while participating and collaborating in building models using heterogeneous data for collaborative perception.

### 4.2. Towards Model-Agnostic Perception Frameworks

Model discrepancies refer to differences or inconsistencies between the perception models used by different vehicles. These discrepancies can arise due to differences in the sensors used by different vehicles, the algorithms employed, or the training data used to train the models. Existing multi-agent perception frameworks have significantly improved 3D object detection and recognition performance. However, they rely on the assumption that every agent working together utilizes the same model with the same parameters and architecture. Especially in AD, it is challenging to put this premise into effect. The sharing of model parameters among AVs can give rise to privacy and confidentiality issues, particularly for AVs developed by different original equipment manufacturers (OEMs). Depending on the type of vehicle and the frequency of model updates, detection models—even for AVs made by the same OEM—can come in different versions. Without properly addressing the discrepancy, the shared sensory data may have a significant domain gap, and the benefit provided by multi-agent perception may quickly wane. Due to the disparity in their confidence scores, different perception models’ performance can suffer. Therefore, to mitigate the detrimental effects of model heterogeneity while ensuring confidentiality, model-agnostic multi-agent perception frameworks must be devised. Furthermore, another potential solution could be using a consensus model that considers the predictions from multiple vehicles.

### 4.3. Heterogeneous and Dynamic Collaboration

Physical occlusion is one of the inescapable barriers to single-node vision; however, these restrictions can be lessened by observing the surroundings from multiple nodes. A potential solution to many current traffic-related problems is the collaboration of vehicles and infrastructure. In order to perform significantly better, vehicle–infrastructure cooperative perception can take advantage of the strengths of both vehicles (that act as mobile perception nodes with lightweight processing power) and infrastructures (that act as fixed nodes but with powerful processing/storage units). To open up a new era of perception for collaborative autonomous driving, it is necessary to develop efficient and dynamic solutions to coordinate information from vehicles with infrastructures. The idea of CP, moreover, is not meant to be limited to a few nodes such as two vehicles or a single vehicle with a single infrastructure. The CP system should be capable of collaborating dynamically with vehicle perception nodes. For instance, the infrastructure perception node must be able to cooperate consecutively with a dynamically varying number of vehicle perception nodes. keeping in view the challenges of CP in real-world development, such as scalability, dynamism, and heterogeneity, a hierarchical structure, integrating vehicles, infrastructure, and Cloud, can be a viable solution. Therefore, developing a unified approach will be a major challenge that can provide a robust base for future studies on cooperative perception.

### 4.4. Value of Information Aware Collaborative Perception

Identifying information that is useful or known to the possible receivers presents one of the biggest challenges in CP. Sharing already-known information could end up being a waste of network resources since each CAV equipped with heterogeneous sensors continues to collect sensor data. A potential solution to this issue is the idea of value-anticipating networking. As a result, each CAV can intelligently foresee the value of every bit of data from the environment surrounding it and messages from other vehicles. Based on the predicted value information, the transmitter vehicle decides which information is communicated, deferred, or even canceled to conserve the network resources and send valuable information more reliably. Vehicles incorporate the information about each observed road object in a message transmission only when an anticipated value exceeds a predetermined threshold for at least one vehicle within the communication range. Furthermore, when determining whether to transmit, the framework should also take visibility and relevancy into account. The network resources are eventually employed for useful information by anticipating the value of information in a distributed fashion; therefore, more solution approaches need to be developed in this area.

### 4.5. Intelligent Context-Aware Fusion

A straightforward solution approach to the AV perception problem is to keep increasing the size and complexity of AV algorithms and putting additional sensors to account for as many driving scenarios as possible. However, the contextual information of the scene is frequently ignored or completely left out of the fusion procedure in dynamic situations; therefore, fusing more sensors may actually produce a less accurate result. Thus, solution approaches that can adapt to dynamically changing driving environments as they emerge without increased computational requirements are essential for robust and accurate AV perception. To maximize robustness without sacrificing efficiency, intelligent context-aware selective sensor fusion frameworks that learn to identify the current driving context, such as weather, lighting, road type, high-level visual features, etc., need to be created. These frameworks will dynamically select the fusion methodology and fuse the best combination of sensors. Selective sensor fusion solution approaches ought to be able to adapt how and when fusion is used on-the-fly.

### 4.6. Towards Multi-Modality Large-Scale Open Datasets

The development of the V2X cooperation dataset will be crucial for the research and validation of V2X cooperation algorithms. Growing large-scale datasets and the advent of deep learning algorithms have enhanced perception performance. Although numerous real-world datasets have been acquired to train models for different perception tasks, the datasets that are currently available within the field of collaborative perception are either of a small scale or are not open to the public. Therefore, further research on CP is precluded by the lack of an open, large-scale dataset. Additionally, high-fidelity simulations are where the majority of datasets are collected. Even though simulation is a cost-effective and secure method of algorithm validation, a realistic dataset is highly required to put CP into practice. The first real-world CP dataset, DAIR-V2X [31], which is a sizable, multi-node, multi-modal CP dataset acquired from both vehicle nodes and infrastructure nodes was released in 2022.

### 4.7. Information Redundancy Mitigation in V2X Collaborative Perception

The ETSI-developed collective perception service (CPS) relies on periodic message exchanges between V2X stations, such as vehicles or roadside units (RSUs), within their communication range. ETSI has standardized the perceived object container (POC), which includes location, speed, and heading information for a specific perceived object. Additionally, ETSI has defined the format of the CPM and established rules for generating and transmitting CPMs. As vehicles continuously generate a significant number of POCs, it is crucial to control their volume to reduce network load while ensuring their usefulness. To address this, ETSI introduced redundancy mitigation schemes that exclude duplicated POCs based on various criteria such as frequency, dynamics, confidence, entropy, object self-announcement, and distance [55]. However, these ETSI schemes have limitations when applied in vehicular environments such as (i) the static thresholds used in these schemes may not perform well in different vehicular environments, and they do not suggest specific values for static thresholds; (ii) the broadcast of POCs does not consider which POCs are useful for each vehicle.

Therefore, to overcome these limitations, it is necessary to develop new approaches. Instead of relying on fixed thresholds and complex formulas, it is possible to eliminate unnecessary perceived object information based on an optimal policy. Aoki et al. [25] introduced a redundancy mitigation scheme based on deep reinforcement learning (DRL), which removes duplicated perceived object information by considering the information perceived by neighboring vehicles. However, this scheme broadcasts perceived object information without treating each vehicle individually, unlike the ETSI schemes. Additionally, since they only addressed data duplication, it becomes challenging to transmit useful perceived object information for vehicular driving. In a similar study, Abdel-Aziz et al. [56] proposed a DRL-based redundancy mitigation scheme that eliminates irrelevant perceived object information for each vehicular driving. They define the usefulness of perceived object information as a reward, taking into account the distance between the location information of the perceived object and the receiving vehicle. Another study by Jung et al. [57] developed a context-aware redundancy mitigation scheme based on DRL, where the usefulness of perceived object information in cyber-physical systems is evaluated using a deep Q-network. To accurately quantify the usefulness of perceived object information for each vehicular driving scenario, the DRL-CARM scheme formulated a reward function considering various vehicular contexts such as location, speed, heading, and perception area based on onboard sensors. It then derived an optimal policy to maximize this reward function. Another challenge in this area is that several rules have been proposed for redundancy mitigation; however, their performance has not been thoroughly evaluated yet. Delooz et al. [58] conducted a comprehensive review, assessment, and comparison of different redundancy mitigation rules, investigating functional and operational requirements. They performed a performance evaluation using discrete-event simulations in a representative city scenario with realistic vehicle densities and mobility patterns.

## 5. Conclusions

Collaborative perception technology is essential for addressing the issues of restricted perception range and insufficient computational power for AVs; however, leveraging wireless communication technology further ensures the real-time requirements of AD. This article comprehensively discusses the transition from classical perception to collaborative perception and perception creation at different levels. Furthermore, a range of crucial challenges and future directions are discussed aiming to assist the industry and researchers in developing a robust and practical evolved version of collaborative perception models.

## Figures and Tables

**Table 1:** A comparison of the characteristics of sensors.

Parameters	Camera	LiDAR	Radar	Ultra-sonic	Fusion and V2X
Field of View	A	A	G	M	G
Edge Detection	G	G	W	W	G
Object Detection	A	A	A	A	G
Distance Estimation	A	G	G	M	G
Object Classification	G	M	A	M	G
Lane Tracking	G	W	W	W	G
Darkness/Light Disturbance	A	G	G	G	G
Visibility Range	A	A	G	M	G
Adverse Weather	M	M	A	M	G
Angular Resolution	G	M	A	W	G
Velocity Resolution	M	G	A	W	G
Range	G	G	G	W	G

**Legend: A**: Acceptable, **G:** Good, **M:** Mediocre, **W:** Worse.

**Table 2:** A comparison of the characteristics of communication technologies.

Feature/ Requirement	DSRC IEEE 802.11p	LTE-V2X	C-V2X	NR-V2X
Technology	Wi-Fi-based	LTE-based	LTE- and 5G-based	5G-based
Communication Range	Up to a few hundred meters	Up to several kilometers	Up to several kilometers	Up to several kilometers
Frequency Band	5.9 GHz	Licensed cellular bands	Licensed cellular bands	Licensed cellular bands
Communication Modes	V2V, V2I, V2P	V2V, V2I, V2P	V2V, V2I, V2P	V2V, V2I, V2P
Direct Communication	Yes	Yes	Yes	Yes
Network Connectivity	No	Yes	Yes	Yes
Latency	Low to medium	Medium to high	Medium to high	Low to medium
Data Rate	Medium to high	High	High	High
Modulation	OFDM	SC-FDMA	SC-FDMA	256 QAM
Security & Privacy	Basic	Enhanced	Enhanced	Enhanced
Spectrum Efficiency	Moderate	High	High	High
Scalability	Limited scalability	High	High	High
Retransmission	No	Yes	Yes	Yes

**Table 3:** Summary of the collaborative perception message-sharing models.

Collaboration Type	Approach	Advantages	Disadvantages	Decision Challenges
Early	Deep Learning	1. A comprehensive understanding of the environment is formed by sharing and collecting raw data. 2. A complete and accurate understanding of the environment enables AVs to make better decisions.	1. Low tolerance for transmission delay and noise, and communication bandwidth is a constraint. 2. Implementing data-level fusion can be expensive as it requires significant computational resources.	These models have decision problems, especially in dynamic environments: 1. Who will decide on which vehicle to provide which data? 2. Who will decide to receive data from which neighboring vehicle? 3. How to intelligently decide on selectively receiving data from the right set of a sensor (camera, LiDAR, radar) from neighboring vehicles. 4. How to decide on the standardized data exchange and fusion process.
Intermediate	Deep Learning	1. High tolerance for noise, delay, and variations in node and sensor models. 2. Reduces the computational demands on individual vehicles. 3. Reduces the complexity of the data and improves the overall perception.	1. Needs training data, and finding a systematic approach to model design is difficult. 2. Features extracted from different sources may be inconsistent, making it difficult to combine them effectively.	
Late	Traditional	1. Simple to develop and implement in real-world systems. 2. Better handling of data heterogeneity.	1. Significantly constrained by incorrect perceptual outcomes or disparities in sources. 2. Can be computationally intensive, requiring significant processing power. 3. Can increase the latency of the system, potentially leading to delays in decision-making.	

## Data Availability

Not applicable.

## Abbreviations

The following abbreviations are used in this manuscript:

ADAS	Advanced Driving Assistance System
AD	Autonomous Driving
AV	Autonomous Vehicle
CAD	Collaborative Autonomous Driving
CAV	Connected and Autonomous Vehicle
CP	collaborative Perception
CPM	Cooperative Perception Message
CPS	Collective Perception Service
CSMA	Carrier Sense Multiple Access
DMS	Dynamic Message Signs
DRL	Deep Reinforcement Learning
DSRC	Dedicated Short-range Communication
eRSU	evolved Roadside Units
ETSI	European Telecommunications Standards Institute
ITS	Intelligent Transport System
POC	Perceived Object Container
OEM	Original Equipment Manufacturers
OFDM	Orthogonal Frequency Division Multiplexing
SAE	Society of Automotive Engineers
URLLC	Ultra-Reliable, Low-Latency Communications
V2I	Vehicle to Infrastructure
V2P	Vehicle to Pedestrian
V2V	Vehicle to Vehicle
V2X	Vehicle to Everything
